# Effect of irradiation on the expression of E-cadherin and β-catenin in early and late radiation sequelae of the urinary bladder and its modulation by NF-κB inhibitor thalidomide

**DOI:** 10.1007/s00066-021-01751-y

**Published:** 2021-03-10

**Authors:** Alexander Krischak, Jakob Kowaliuk, Sina Sarsarshahi, Wolfgang Dörr, Miriam Kleiter

**Affiliations:** 1 Platform Radiooncology and Nuclear Medicine, Department for Companion Animals and Horses, University of Veterinary Medicine of Vienna, Vienna, Austria; 2 ATRAB-Applied and Translational Radiobiology, Department of Radiation Oncology, Medical University of Vienna, Vienna, Austria

**Keywords:** Adherens junction proteins, Nuclear factor kappa B, Urothelial barrier, Radiation cystitis, Radiotherapy, Thalidomide

## Abstract

**Purpose:**

In a previous study we have shown in a mouse model that administration of nuclear factor-kappa B (NF-κB) inhibitor thalidomide has promising therapeutic effects on early radiation cystitis (ERC) and late radiation sequelae (LRS) of the urinary bladder. The aim of this study was to evaluate in the same mice the effect of thalidomide on adherens junction (AJ) proteins in ERC and LRS.

**Methods:**

Urothelial expressions of E‑cadherin and β‑catenin were assessed by immunohistochemistry in formalin-fixed paraffin-embedded (FFPE) bladder specimens over 360 days post single-dose irradiation on day 0. First, the effect of irradiation on AJ expression and then effects of thalidomide on irradiation-induced AJ alterations were assessed using three different treatment times.

**Results:**

Irradiation provoked a biphasic upregulation of E‑cadherin and β‑catenin in the early phase. After a mild decrease of E‑cadherin and a pronounced decrease of β‑catenin at the end of the early phase, both increased again in the late phase. Early administration of thalidomide (day 1–15) resulted in a steeper rise in the first days, an extended and increased expression at the end of the early phase and a higher expression of β‑catenin alone at the beginning of the late phase.

**Conclusion:**

Upregulation of AJ proteins is an attempt to compensate irradiation-induced impairment of urothelial barrier function. Early administration of thalidomide improves these compensatory mechanisms by inhibiting NF-κB signaling and its interfering effects.

## Introduction

Radiotherapy is an essential modality of cancer treatment that is applied to around 50% of human cancer patients in the form of a primary, adjuvant or neoadjuvant therapy [1, 2]. Despite high precision tumor targeting achieved by modern radiotherapy (by intensity-modulated radiation therapy, image-guided treatments, etc.), normal tissues adjacent to the tumor are exposed to a radiation dose which is associated with adverse effects [3, 4]. An organ at risk during pelvic radiotherapy is the urinary bladder and two phases of an inflammatory response are reported to occur after exposure [5]. Early symptoms are described as early radiation cystitis (ERC) and they are characterized by an increased miction rate correlating with a reduction in urinary bladder storage capacity, dysuria, nycturia and hematuria. ERC occurs days to weeks after the start of radiotherapy and usually resolves within several weeks, treated only symptomatically to alleviate clinical symptoms [5–7]. After a dose-dependent, symptom-free latency period, which can last for several months or up to decades, a progressive and irreversible chronic phase occurs, often referred to as late radiogenic sequelae (LRS) of the bladder (also referred to as hemorrhagic cystitis or radiation cystitis), for which no standard treatment guidelines are available [5–8]. Besides clinical symptoms similar to the ERC, the late phase is characterized by fibrotic changes of the bladder wall leading to an enormous loss of bladder volume and urinary incontinence due to detrusor dysfunction [9].

Currently, the only possibility to decrease the severity of LRS is a reduction of the radiation dose, resulting in lower tumor control and survival rate [10]. In order to increase the patient’s quality of life with the same tumor control rate, treatment methods for LRS are urgently needed. LRS in the urinary bladder are directly modified by severity and duration of early response reactions, known as consequential late effects (CLE) [11]. Presumably, radiation-induced impairment of the protective barrier function of the urothelial wall leads to additional injury of the underlying tissue by permeating urine, which increases the severity of the late effects [8, 12, 13]. The adherens junction (AJ) proteins E‑cadherin and β‑catenin are important for epithelial barrier function [14, 15]. E‑cadherin is a calcium-dependent cell–cell adhesion molecule, which is linked by β‑catenin and other proteins to the actin cytoskeleton [14]. Radiation-induced adverse effects on the E‑cadherin/β-catenin complex have already been discovered in different tissues [16]. In addition, E‑cadherin/β-catenin are involved in fibrotic diseases of, for example, lung or kidney [14].

Present treatment methods for ERC did not show any ameliorating effects on LRS, but current research data suggested that the nuclear factor-kappa B (NF-κB) pathway is involved in the inflammatory processes [17]. Functional studies in the same mouse model as in the current study assessed the time course of ERC and LRS by transurethral cystotonometry and immunohistochemical analyses and showed a biphasic activation of the NF-κB proteins p50 and p65 during ERC and p50 reactivation in the late phase [17]. Furthermore, the systemic administration of the NF-κB inhibitor thalidomide during the early phase had promising beneficial effects on ERC and the incidence and severity of the LRS [17]. It reduced NF-κB activation and shifted the ED50 value for early radiation cystitis and late radiation sequelae to higher doses [17]. Mitigating effects of thalidomide in radiation-induced lung fibrosis had been discovered as well [19].

The aim of the present study was to investigate as a follow-up to our previous study [17] the effects of irradiation on the AJ proteins E‑cadherin and β‑catenin and their modulation by the NF-κB inhibitor thalidomide. The urothelial expression of E‑cadherin and β‑catenin was investigated in single-dose experiments in the same mice with a follow-up of 360 days.

## Materials and methods

### Animals and housing

The present study used archived formalin-fixed paraffin-embedded (FFPE) bladder specimens of female C3H/Neu mice. The samples had been collected in our previous study that investigated NF-κB modulation by thalidomide on early and late radiation sequelae in urinary bladder dysfunction by assessment of NF-κB proteins p50/p65 and functional cystotonometry [17].

Detailed information about animals and housing is provided in this previous study [17]. Briefly, five to six mice were housed per cage under specific pathogen-free conditions. All animal experiments were performed in accordance with the current laboratory animal welfare legislation with approval of the respective authorities (Federal Ministry of Education, Science and Research, file number BMWF-66.009/0148-WF/II/3b/2014).

### Irradiation

The technique of pelvic irradiation of mice was described in detail in several previous studies [8, 17, 18, 20]. Briefly, a single dose of 23 Gy was prescribed to the emptied bladder of anesthetized mice with a YXLON MG325 X‑ray device (YXLON International GmbH, Hamburg, Germany), operating at 200 kV and 20 mA. The selected dose of 23 Gy corresponds to the dose causing functional bladder impairment in 90% of irradiated animals. Functional response was defined by a > 50% reduction of pre-irradiated urinary bladder compliance at 10 mm Hg intravesicular pressure assessed by transurethral cystotonometry as described in our previous study [17].

### Experimental design

A total of 915 mice were assigned to the following five groups (Table 1). A non-irradiated control group served as age-related control and was neither irradiated nor treated with thalidomide (216 samples). An irradiation control group was irradiated on day 0 (216 samples). Three thalidomide (THA) treatment groups were irradiated on day 0 and further treated with thalidomide. Thalidomide was administered intraperitoneally (100 mg/kg thalidomide dissolved in 80% dimethyl sulfoxide (DMSO), injected volume not exceeding 150 µL) either daily from day 1 to 15 in treatment group THA1–15 (216 samples) or from day 16 to 30 in treatment group THA16–30 (141 samples) or in two-day intervals from day 150 to 180 in treatment group THA150–180 (126 samples) [17].

**Table 1:** Summary of the experimental design

Treatment groups	Irr	THA	Number of samples			
			d1–15	d16–30	d60–360	Total
*Non-irradiated control*	None	None	75^a^	75^a^	66^b^	216
*Irradiation control*	d0	None	75^a^	75^a^	66^b^	216
*THA1–15*	d0	Daily d1–15	75^a^	75^a^	66^b^	216
*THA16–30*	d0	Daily d16–30	None	75^a^	66^b^	141
*THA150–180*	d0	EOD 150–180	None	None	126^c^	126

*d* day, *THA* thalidomide, *Irr* irradiation, *EOD* every other day (EOD)

^a^5 samples per day

^b^6 samples every 30 days

^c^6 samples EOD d150–180 and 6 samples every 30 days d210–360

### Immunohistochemical analyses and sample preparation

Paraformaldehyde (Roti®-Histofix 4% acid-free, ph 7, Roth [Carl Roth GmbH + Co KG, Karlsruhe, Baden-Württemberg, Germany]) was instilled to the bladders by transurethral catheter and excised bladders were further immersed in 4% paraformaldehyde for 48 h. This combination of perfusion fixation and immersion fixation was performed to fixate the tissue from both sides rapidly [21]. After 48 h incubation at room temperature the bladders were cut into sagittal halves, embedded in paraffin and stored at 4 °C.

Tissue sections of 4 µm were cut by a rotary microtome (Leica Biosystems Nussloch GmbH, Nussloch, Baden-Württemberg, Germany) and tissue sections of two bladders per adhesive microscope slide were fixed with an incubator (BD115, Binder GmbH, Tuttlingen, Germany) overnight. Twenty slides were taken to rehydration and to standard immunohistochemistry staining procedures at once. For antigen retrieval, all samples were boiled in citrate buffer (pH 6) for 20 min by microwave (600 W). The primary antibodies were purchased from Abcam® (Cambridge, MA, USA). Preliminary staining experiments were performed to determine the optimal antibody dilutions in order to clearly visualize constitutively expressed E‑cadherin and β‑catenin in the non-irradiated control group as well as to easily detect differences in staining intensity in comparison to the control group. A final dilution of 1:500 with Tris-buffered saline was used for E‑cadherin antibody (ab76319, rabbit monoclonal) and β‑catenin antibody (ab32572, rabbit monoclonal). Samples were incubated with the antibody dilution at 4 °C overnight. The Vectastain® ABC rabbit kit (Vectastain® ABC Kit, Peroxidase (Rabbit IgG), Vector Laboratories, Burlingame, CA, USA) and 3,3-diaminobenzidine (DAB) chromogen (ImmPact® DAB Substrat Kit, Peroxidase, Vector Laboratories, Burlingame, CA, USA) were used for signal amplification and visualization. Hematoxylin was used for unspecific background staining. All slides were fixated with Entellan (Entellan®new, Merck KGaA, Darmstadt, Germany) to preserve the tissue.

Immunohistochemical analysis was performed with a light microscope (Olympus Europa SE&CO. KG, Hamburg, Germany) at 400× and 800× magnification. The staining intensity of the urothelial cell walls was rated with a score from 0 to 3. Five sample-representing optical fields at 800× magnification were used to determine a final score. Score 1 was defined as a moderate membranous staining of the urothelial cell walls. Score 0 was defined as a clearly visible loss of intensity compared to score 1. Samples with a score of 2 showed strong membranous staining of the cell walls. Score 3 was defined as strong staining intensity and thickening of all urothelial cell walls, often accompanied with a strong cytoplasmic staining.

### Statistical analysis

Statistical analysis and graphical formatting were performed by SPSS Statistics for Windows (Version 26, IBM, Corp., Armonk, NY, USA) and GraphPad Prism 5 (Version 5.03, GraphPad Software, Inc., La Jolla, CA, USA).

Statistically significant differences between treatment groups and sampling days were calculated by two-way analysis of variance (ANOVA) and Tukey-HSD (Honest Significant Difference) post-testing for all groups. First, the irradiated control group was compared with the non-irradiated control to assess the effect of irradiation on E‑cadherin and β‑catenin expression. Then the irradiation control group was compared with the different thalidomide treatment groups to analyze the effects of thalidomide at different treatment times on the radiation-induced modulation of both AJ proteins. A *p*-value of < 0.05 was considered as statistically significant.

## Results

### Effect of irradiation on E-cadherin and β-catenin expression

**Fig. 1:**
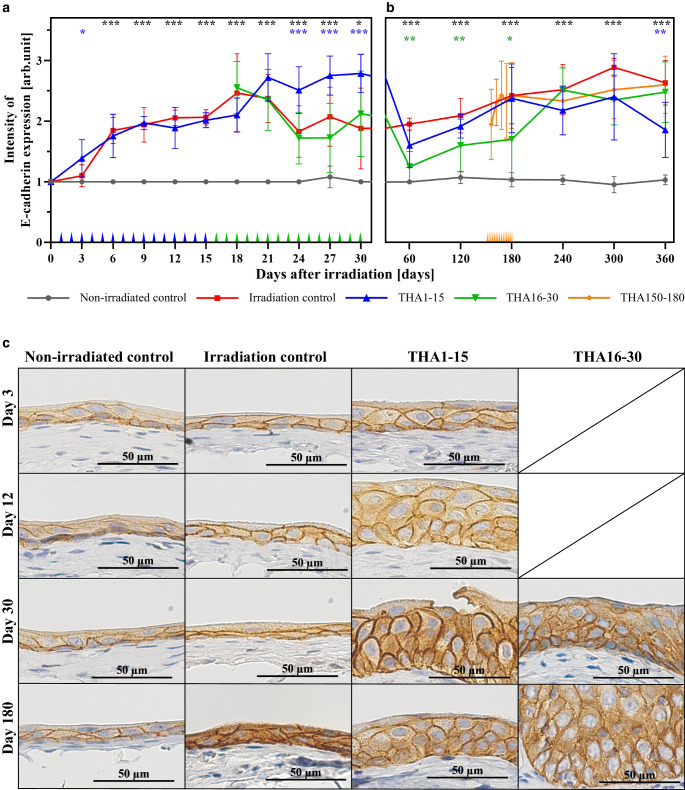
Effect of irradiation at day 0 on E‑cadherin expression and its modulation by thalidomide (THA): **a** Early phase: Data points at 3‑day intervals represent the average immunohistochemical intensity (IHC) score from 15 samples (5 samples per day). **b** Late phase: Data points at 60-day intervals represent the average IHC score from 12 samples (6 samples every 30 days). Additional data points at 6‑day intervals are shown in the THA150–180 group and represent the average IHC score from 18 samples (6 samples every other day). Significant differences between THA treatment groups and irradiated control are marked with *stars* in the *color* of the respective treatment group. Different THA treatment times are marked in the *color* of the respective group along the x‑axis (daily application in THA1–15 and THA16–30; every second day in THA150–180). **c** Representative photomicrographs of urothelium at 400× magnification at day 3, 12, 30 and 180 demonstrate changes in intensity of immunoreactivity for E‑cadherin. *p ≤ 0.05; **p ≤ 0.01; ***p ≤ 0.001; scale bar = 50 µm

**Fig. 2:**
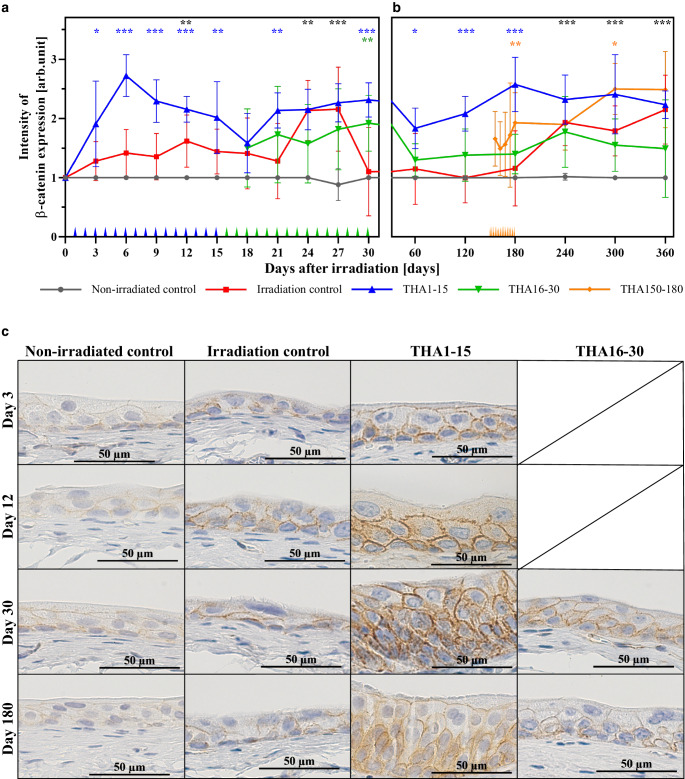
Effect of irradiation at day 0 on β catenin expression and its modulation by thalidomide (THA): **a** Early phase: Data points at 3‑day intervals represent the average immunohistochemical intensity (IHC) score from 15 samples (5 samples per day). **b** Late phase: Data points at 60-day intervals represent the average IHC score from 12 samples (6 samples every 30 days). Additional data points at 6‑day intervals are shown in the THA150–180 group and represent the average IHC score from 18 samples (6 samples every other day). Significant differences between THA treatment groups and irradiated control are marked with *stars* in the *color* of the respective treatment group. Different THA treatment times are marked in the *color* of the respective group along the x‑axis (daily application in THA1–15 and THA16–30; every second day in THA150–180). **c** Representative photomicrographs of urothelium at 400× magnification at day 3, 12, 30 and 180 demonstrate changes in intensity of immunoreactivity for β catenin. *p ≤ 0.05; **p ≤ 0.01; ***p ≤ 0.001; scale bar = 50 µm

Initially, we compared the irradiated control group (216 samples) with the non-irradiated control group (216 samples) to determine the radiogenic effect on the expression of E‑cadherin and β‑catenin. In the non-irradiated control both AJ proteins were constitutively expressed with a score of approximately 1 over the whole study period of 360 days. As a result, alterations of the expression levels of E‑cadherin and β‑catenin by single dose irradiation could be clearly visualized (Figs. 1 and 2).

#### Early phase

E‑cadherin expression started to rise on day 3 and reached a first plateau from day 6 to day 15 with a significant difference in expression levels compared to the non-irradiated control (Fig. 1a, c). Then E‑cadherin expression increased to a second higher peak from day 18 to day 21. From day 24 to day 30 E‑cadherin expression decreased to values comparable with the first plateau.

Also, β‑catenin expression started to rise on day 3 and a flattened plateau was seen between day 6 to day 18 with a small peak on day 12. Only this peak value reached a significant difference in comparison to β‑catenin expression in the non-irradiated group (Fig. 2a, c). A second pronounced plateau occurred from day 24 to day 27 with a significant difference to the non-irradiated control. At day 30 expression levels decreased nearly to constitutive levels.

#### Late phase

E‑cadherin expression increased continuously in the late phase post irradiation and reached a plateau from day 180 to 360 with a peak value at day 300. A statistically significant difference between the irradiated control and the non-irradiated control was observed for the entire late phase from day 60 to 360 (Fig. 1b, c).

The expression of β‑catenin remained at low, nearly constitutive levels like in the non-irradiated control group until day 180. Then β‑catenin expression increased to a plateau from day 240 to 360 with significantly higher expression levels in comparison to the non-irradiated control (Fig. 2b, c).

### Effect of thalidomide on radiation-induced AJ alterations

Our initial study indicates that early administration of the NF-κB inhibitor thalidomide has ameliorating effects on ERC and LRS and that the NF-κB signaling cascade is involved in the inflammatory process [17]. We suggest that the NF-κB signaling cascade is negatively interfering with mechanisms that try to compensate the radiation-induced impairment of the urothelial barrier function, like the upregulation of the AJ proteins E‑cadherin and β‑catenin. Therefore, we compared the irradiation control group with the treatment groups that received irradiation and thalidomide at different treatment times.

#### Early phase

In the early thalidomide treatment group (THA1–15) the first increase in E‑cadherin expression started steeper in comparison to the irradiation control with a significant higher level on day 3. From day 6 to day 15 E‑cadherin expression showed a similar plateau as in the irradiated-control without a significant difference. Then, the second higher plateau occurred slightly later (day 21 instead day 18) and remained over an extended period of time until the end of the early phase (day 21–30 instead of day 18–21), leading to significantly higher E‑cadherin levels from day 24 to day 30 (Fig. 1a, c).

Thalidomide administration in the second early phase (THA16–30) had no significant effect on E‑cadherin expression levels in comparison to the irradiation control group from day 18 to 30 (Fig. 1a, c).

Early thalidomide application (THA1–15) also caused a steeper increase in β‑catenin expression in comparison to the irradiation control with a pronounced peak on day 6. High β‑catenin levels were observed throughout the entire flattened plateau phase of the irradiated control until day 15, resulting in significant higher expression levels from day 3 to day 15. On day 18 β‑catenin expression dropped to similar levels as in the irradiation control, followed by a rise into an earlier and extended second plateau (day 21–30 instead of day 24–27) with significant higher β‑catenin levels on day 21 and day 30 (Fig. 2a, c).

Thalidomide administration in the second early phase (THA16–30) resulted in a broader but lower second β‑catenin plateau in comparison to the irradiation control (day 21–30 instead of day 24–27) with a significant difference only on day 30 (Fig. 2a, c).

#### Late phase

With the beginning of the late phase on day 60 E‑cadherin expression in the THA1–15 group dropped to levels slightly below levels of the irradiation control with no significant difference from day 60 to day 300. Only at the end of the late phase on day 360 E‑cadherin expression was significantly lower than in the irradiation control (Fig. 2b).

In the THA16–30 group E‑cadherin expression levels also dropped at the beginning of the late phase below the irradiation control with significantly lower levels from day 60 to 180. Then E‑cadherin expression increased to a plateau from day 240 to 360 without any significant difference to the irradiation control (Fig. 2b).

In the late thalidomide group (THA150–180) E‑cadherin expression showed a parallel, slightly lower time curve than the irradiation control without any significant differences from day 180 to 360 (Fig. 2b).

In the early thalidomide treatment group (THA1–15) β‑catenin expression at the beginning of the late phase had dropped to lower levels than during the end of the early phase. From day 60 to 180 expression levels increased continuously and reached a peak on day 180 with significantly higher expression levels than the irradiation control over the entire time. From day 240 to 360 expression levels showed a comparable slightly higher plateau than the irradiation control without any significant difference.

In the THA16–30 group β‑catenin expression levels showed no significant difference to the irradiation control over the entire late phase. The β‑catenin expression dropped to a low level on day 60 and remained low until day 180 with values that were slightly higher than in the irradiation control. Then the expression increased to a comparable, slightly lower plateau from day 240 to 360 than the irradiation control (Fig. 2b, c).

In the THA150–180 group the β‑catenin expression increased in two steps with significantly higher expression levels on day 180 and 300 than the irradiation control (Fig. 2b, c).

## Discussion

The present study investigated the effect of irradiation on the expression of E‑cadherin and β‑catenin in the urothelial wall and its modulation by NF-kB inhibitor thalidomide in a mouse model.

The E‑cadherin/β-catenin complex is crucial for epithelial barrier integrity and studies in intestinal epithelium of mice and in the dermis of a mouse footpad model suggest that radiation can harm AJ [16, 22]. In the mouse footpad model two distinctive phases of the damage response were identified [16]. An early phase with loss of E‑cadherin mediated cell contact, and a regenerative phase, during which Wnt/β-catenin signaling was activated [16]. For the mouse urothelium a biphasic early radiation response phase from day 0–15 (early phase 1) and from day 16–30 (early phase 2), followed by a late response phase is well characterized [8, 17, 18, 23, 24], but information about changes in AJ protein expression is not available. Therefore, we investigated the effect of irradiation on E‑cadherin and β‑catenin in the urothelium during ERC and LRS. In contrast to skin epidermis, urothelium is not characterized by a fast cell turn-over and no breakdown of cell contacts is described during the early radiation response [8]. However, it is known that total cell number of the urothelium decreases in the early phase 1 to a first nadir on day 13, followed by slight recovery at the beginning of the early phase 2 and a second and more pronounced nadir at the end of the early phase [25]. The most pronounced cell loss is observed in the superficial layer with a progressive reduction of umbrella cells accompanied by a loss of luminal uroplakin III until the end of the early phase [8]. Results of our study suggest a compensatory induction of AJ to maintain and re-establish urothelial barrier integrity and counteract urothelial cell loss and damage to the permeability layer. A first increase in both AJ occurred during the first early phase followed by a stronger upregulation in the second early phase. The time course of E‑cadherin and β‑catenin upregulation is comparable to the time course of the functional bladder response [17]. In our previous study in the same mice functional bladder impairment reached a peak in the ERC around day 18 to 21 post irradiation [17]. E‑cadherin expression reached a peak around day 18 to 21, like the peak in early functional bladder response [17]. The β‑catenin expression showed a peak around day 24 to 27. Our results are consistent with human studies that report elevated E‑cadherin levels in the bladder urothelium after long-term exposure to low-dose ionizing radiation as well as in patients with interstitial cystitis [26, 27].

Parallel to the direct loss of urothelial cells, an inflammatory response is induced by irradiation that contributes to the clinical manifestation of ERC [17, 23, 25]. NF-κB signaling is involved in this inflammatory response [17]. In our previous study, we demonstrated a biphasic activation of the NF-κB proteins p50 and p65 in the urothelium of the same mice as in the current study during the early radiation response [17]. P50/p65 heterodimers are known for the induction of inflammatory genes [28, 29]. The NF-κB-inhibitor thalidomide can reduce activation of p50 and p65 as demonstrated in our previous study [17]. Early administration of thalidomide lowered the activation of p50 and p65, reduced the incidence of animals with functional bladder impairment in the early phase and significantly increased the ED50 dose for ERC [17]. The therapeutic effect of thalidomide was most pronounced in the THA1–15 group [17]. In our current study thalidomide treatment in the early phase 1 (THA1–15) resulted in an increased expression of E‑cadherin and β‑catenin at the cell membrane. Especially at the beginning of the early phase 1 a significantly steeper rise was observed and this effect was more pronounced for β‑catenin. In the early phase 2, a significantly higher and extended plateau was noticed in comparison to the irradiation control. Multifaceted crosstalk between NF-κB and Wnt/β-catenin as well as NF-κB and E‑cadherin has been described in the context of inflammation, proliferation and neoplastic disease [30–42]. Direct or indirect inhibition of NF-κB can upregulate E‑cadherin and co-localization with β‑catenin [31–36, 38]. Also, COX‑2 can influence E‑cadherin expression through NF-κB and irradiation-induced upregulation of COX‑2 in the ERC has been shown in the same mouse model in an older study [23, 36]. It seems that inhibition of NF-κB represses acute inflammatory signals on E‑cadherin/β-catenin and therefore indirectly enhances the postulated compensatory effects to stabilize urothelial barrier integrity, for which adherens junction assembly is crucial [14, 15]. Consistent with our findings, radiation-induced dermatitis was ameliorated in a rodent model by reducing E‑cadherin/β-catenin degradation by genistein or dasatinib treatment [16]. Consistent with functional data of our previous study, administration of thalidomide in the early phase 2 did not achieve the same significant modulation of E‑cadherin and β‑catenin, most likely because the initial inflammatory cascade is not interrupted. Repression of the initial of inflammation via NF-κB inhibition and subsequent elevation of E‑cadherin has also been reported in the treatment of pulmonary fibrosis [35]. Further, an early treatment time point was also essential to achieve an ameliorating effect on the early bladder response with the human keratinocyte growth factor palifermin in the same mouse model [43].

At the beginning of the late phase clinical signs of ERC have resolved. However, a depletion of total urothelial cell number, especially superficial umbrella cells and reduced luminal uroplakin III is still present at that time, indicating long lasting radiation-induced damage to the urothelium with low compensatory potential [8, 18, 23, 25]. Restoration of the urothelium occurs around day 180 and ongoing proliferative activity results in urothelial hyperplasia at the end of the late phase [25]. In our study, only E‑cadherin but not β‑catenin showed significantly higher expression levels at the beginning of the late phase until day 180 compared to the non-irradiated control. Thereafter a radiation-induced upregulation was seen for both AJ proteins until the end of the late phase. Functional data from our previous study demonstrated a mean complication-free survival of 166 days prior to clinical onset of LRS [17].

Furthermore, only NF-κB protein p50 but not p65 was activated during the late phase in our previous study [17]. P50 homodimers are associated with the upregulation of driving factors for chronic inflammation [17, 28, 44–48]. Thalidomide treatment during this late phase (THA150–180) achieved no relevant treatment effect in our previous functional study and it seems that thalidomide has no long-lasting effects on chronic NF-κB activation [17]. Thalidomide treatment during the late phase (THA150–180) had also no significant effect on E‑cadherin expression and only minor effects on β‑catenin with an earlier rise on day 180 and a higher expression on day 300 than in the irradiated control.

However, treatment with thalidomide during the early radiation response revealed a significant therapeutic effect on LRS with an extended median complication-free survival time of 300 days and a significantly higher ED50 dose [17]. Severity of radiation response in early phase 2 has been shown to correlate significantly with the development of a late response and is itself influenced by the severity of the first acute wave, suggesting a significant consequential component [18]. Depletion of the bladder urothelium and damage to the permeability barrier is most pronounced at the end of the early phase, resulting in leakage of hyperosmolar urine into deeper subepithelial connective tissues as well as muscle layers [8]. In our study, early thalidomide treatment (THA1–15) resulted in an extended upregulation at the end of the early phase 2 for both AJ proteins. This effect was especially pronounced for E‑cadherin. Furthermore, a significant increase of β‑catenin in the beginning late phase from day 60 to 180 was observed, whereas no significant effect was seen for E‑cadherin. We suggest that thalidomide is indirectly improving the urothelial barrier function by inhibiting negatively interfering effects of NF-κB on AJ, resulting in enhanced expression of membranous E‑cadherin and β‑catenin at the end of the early response phase. Further, it seems that at the beginning of the late phase, thalidomide additionally influences β‑catenin independently from E‑cadherin. It is suggested that thalidomide potentially supports recovery and regeneration of the urothelium in the beginning late phase by upregulation of Wnt/β-catenin. Wnt/β-catenin and Hippo signaling is also reported in the recovery phase of radiation-induced dermatitis [16]. Furthermore, the pronounced increase of β‑catenin in the early phase 1 in the THA1–15 group might indicate active Wnt/β-catenin additionally to upregulation of AJ proteins. Unfortunately, nuclear β‑catenin was not visualized in our study. It seems difficult to assess nuclear β‑catenin in the urothelium by immunohistochemistry. Other studies in normal, inflamed and neoplastic urothelium were also not able to detect nuclear β‑catenin staining and β‑catenin remained membranous or cytoplasmic [49, 50]. Future studies are needed to confirm activated Wnt/β-catenin in the recovery phase of the urothelium.

Limitations of this study were that alterations in AJ protein expression was only assessed by immunohistochemistry in FFPE tissue specimens. Further, DMSO was used to dissolve the non-water-soluble thalidomide for intraperitoneal injection as recommended by the manufacturer. DMSO itself has anti-inflammatory potential via NF-κB inhibition [51]. Therefore, we cannot exclude a small additional effect from the dissolvent itself on the therapeutic effect of thalidomide. Further, the treatment effect of thalidomide in irradiated animals was evaluated only in comparison to an irradiation control. We did not analyze whether constitutively expressed E‑cadherin and β‑catenin in the non-irradiated control is altered by thalidomide because we did not expect an effect in the absence of any NF-κB activation. However, to better understand the complex interactions between thalidomide and the multifaceted signaling pathways that are involved in the pathogenesis of radiation-induced urinary bladder dysfunction, further studies including modern high-throughput methods such as RNA microarray technology, next generation sequencing, mass spectrometry or western blot analyses are warranted.

## Conclusion

The present study revealed that compensatory mechanisms during early radiation cystitis (ERC) and late radiation sequelae (LRS) increase the E‑cadherin and β‑catenin expression to maintain or re-establish the integrity of the urothelial barrier function. Early administration of thalidomide improves these mechanisms by inhibiting the nuclear factor-kappa B (NF-κB) signaling cascade and its interfering effects. As a result, a steeper rise of both adherens junction (AJ) proteins was found in the first few days post irradiation together with a higher and extended expression in the important second early phase, which is crucial for the severity of LRS. Findings of the current and the previous study indicate that thalidomide has to be administered at the beginning of the early phase to achieve beneficial effects. Future studies are needed to confirm activated Wnt/β-catenin in the recovery phase of the urothelium.
